# Quantification of cellular volume and sub-cellular density fluctuations: comparison of normal peripheral blood cells and circulating tumor cells identified in a breast cancer patient

**DOI:** 10.3389/fonc.2012.00096

**Published:** 2012-08-09

**Authors:** Kevin G. Phillips, Anand Kolatkar, Kathleen J. Rees, Rachel Rigg, Dena Marrinucci, Madelyn Luttgen, Kelly Bethel, Peter Kuhn, Owen J. T. McCarty

**Affiliations:** ^1^Department of Biomedical Engineering, School of Medicine, Oregon Health and Science UniversityPortland, OR, USA; ^2^Cell Biology Department, The Scripps Research Institute, La JollaCA, USA; ^3^Scripps Clinic Medical Group, Scripps Clinic, La JollaCA, USA; ^4^Department of Cell and Developmental Biology, School of Medicine, Oregon Health and Science UniversityPortland, OR, USA

**Keywords:** circulating tumor cells, breast cancer, differential interference contrast microscopy, cellular volume, cellular density

## Abstract

Cancer metastasis, the leading cause of cancer-related deaths, is facilitated in part by the hematogenous transport of circulating tumor cells (CTCs) through the vasculature. Clinical studies have demonstrated that CTCs circulate in the blood of patients with metastatic disease across the major types of carcinomas, and that the number of CTCs in peripheral blood is correlated with overall survival in metastatic breast, colorectal, and prostate cancer. While the potential to monitor metastasis through CTC enumeration exists, the basic physical features of CTCs remain ill defined and moreover, the corresponding clinical utility of these physical parameters is unknown. To elucidate the basic physical features of CTCs we present a label-free imaging technique utilizing differential interference contrast (DIC) microscopy to measure cell volume and to quantify sub-cellular mass-density variations as well as the size of subcellular constituents from mass-density spatial correlations. DIC measurements were carried out on CTCs identified in a breast cancer patient using the high-definition (HD) CTC detection assay. We compared the biophysical features of HD-CTC to normal blood cell subpopulations including leukocytes, platelets (PLT), and red blood cells (RBCs). HD-CTCs were found to possess larger volumes, decreased mass-density fluctuations, and shorter-range spatial density correlations in comparison to leukocytes. Our results suggest that HD-CTCs exhibit biophysical signatures that might be used to potentially aid in their detection and to monitor responses to treatment in a label-free fashion. The biophysical parameters reported here can be incorporated into computational models of CTC-vascular interactions and *in vitro* flow models to better understand metastasis.

## Introduction

Cancer metastasis, the leading cause of cancer-related deaths, is facilitated in part by the hematogenous transport of circulating tumor cells (CTCs) from the primary tumor site to distant organs. Though CTCs circulate in exceedingly small quantities, approximately 1 CTC per 10^9^ blood cells, clinical studies have demonstrated that CTCs circulate in the blood of patients with metastatic disease across all major types of carcinomas (Allard et al., 2004), and that the number of CTCs in peripheral blood is correlated with overall survival in metastatic breast (Cristofanilli et al., 2004), colorectal (Cohen et al., 2008) prostate (Scher et al., 2009) and non-small cell lung (Nieva et al., 2012) cancer, while case reports suggest that CTCs possess morphological features present in corresponding primary and/or metastatic lesions in breast (Marrinucci et al., 2007), colorectal (Marrinucci et al., 2009a), and lung (Marrinucci et al., 2009b) cancer. Together, these studies indicate that CTCs can be used to survey primary and metastatic lesions through minimally-invasive peripheral blood draws.

To date, label-based microscopy techniques have been instrumental in identifying CTCs and characterizing the CTC phenotype. Putative CTCs in existing purification assays are typically identified using immunofluorescent antibody labels to epithelial (EpCAM, CK) and leukocyte (CD45) cell surface markers as well as fluorescent nuclear (DAPI) staining to differentiate CTCs and leukocytes based on fluorescence expression profiles: CD45−CK+DAPI+ (CTC) vs. CD45+CK−DAPI+ (leukocyte) (Racila et al., 1998; Vona et al., 2000; Krivacic et al., 2004; Hsieha et al., 2006; Nagrath et al., 2007). The combined use of fluorescent antibodies to cell surface markers and bright field microscopy based stains (Papanicolau, Wright-Giemsa), for labeling of the nuclear and cytoplasmic cellular compartments, has been central in establishing the pleomorphic similarity of CTCs to their corresponding primary and/or metastatic lesions (Marrinucci et al., 2007, 2009a,b). While there is great potential to monitor metastasis through CTC enumeration and qualitative investigations of their morphology using these label-based methods, the basic physical features (e.g., mass, volume, density, density fluctuations), discernable through label-free optical methods, of CTCs remain ill defined. Moreover, the corresponding clinical utility of these physical parameters is unknown.

To elucidate the basic physical features of CTCs we developed a label-free microscopy technique utilizing differential interference contrast (DIC) to quantitatively elucidate cell volume, sub-cellular mass-density variations, and the average size of subcellular constituents inferred from spatial mass density correlations. We report volume, mass density fluctuations, denoted σ_*A*_ [−], and mass density spatial correlations, denoted *L*_*C*_ [μm], for CTCs isolated from a metastatic breast cancer patient using the HD-CTC assay (Marrinucci et al., 2012). The physical properties of HD-CTCs are compared across the normal cellular constituents of blood: platelets (PLT), red blood cells (RBCs), and leukocytes.

## Materials and methods

### HD-CTC and leukocyte identification and characterization

A 54-year-old breast cancer patient provided informed consent at Scripps Clinic (La Jolla, CA) as approved by the Institutional Review Board. The patient presented in October 2007 with bilateral invasive ductal mammary carcinoma and biopsy-proven metastatic disease to bone. The right breast was ER/PR+/HER-2−, while the left breast was ER/PR/HER-2+ with a positive axillary node by fine needle aspiration. A bony site biopsy was ER+, PR−, and HER-2+, all by immunohistochemistry. Blood was taken prior to a bilateral mastectomy in March 2010.

At each draw, 8 mL of peripheral blood was collected in a rare cell blood collection tube (Streck, Omaha, NE) and processed within 24 h after phlebotomy. CTCs were identified using the HD-CTC method, the sensitivity, and specificity of which has been previously reported in Marrinucci et al. (2012). Briefly, the HD-CTC isolation and characterization technique consists of a RBC lysis, after which nucleated cells are attached as a monolayer to custom-made glass slides. Slides are subsequently incubated with antibodies against cytokeratins (CK) 1, 4–8, 10, 13, 18, and 19; and CD45 with Alexa 647-conjugated secondary antibody, nuclei were counterstained with DAPI. For HD-CTC identification, an automated digital fluorescence microscopy technique was used to identify putative HD-CTCs. Fluorescence images of CTC candidates were then presented to a hematopathologist-trained technical analyst for interpretation. Cells are classified as HD-CTCs if they are CK-positive, CD45-negative, contained an intact DAPI nucleus without identifiable apoptotic changes or a disrupted appearance and were morphologically distinct from surrounding leukocytes. Leukocytes were classified according to a CK-negatiave, CD45-positive, DAPI-positive fluorescence status. Cartesian coordinates for each HD-CTC on a slide are generated from a fixed fiduciary marking and used to relocate the cells of interest for DIC measurements. Leukocytes located in the same field of view of HD-CTCs were selected at random to be quantitatively compared to the HD-CTC population.

### Preparation of human platelets

Human venous blood was drawn from healthy donors into citrate-phosphate-dextrose (1:7 vol/vol). PLT rich plasma (PRP) was prepared by centrifugation of anticoagulated blood at 200 g for 10 min. PLTs were further purified from PRP by centrifugation at 1000 g in the presence of prostacyclin (0.1 μg/mL). Purified PLTs were resuspended in modified HEPES/Tyrode buffer (129 mM NaCl, 0.34 mM Na_2_HPO_4_, 2.9 mM KCl, 12 mM NaHCO_3_, 20 mM HEPES, 5 mM glucose, 1 mM MgCl_2_; pH 7.3) containing 0.1 μg/mL prostacyclin. PLTs were washed once by centrifugation and resuspended in modified HEPES/tyrode buffer at indicated concentrations. Purified PLTs were fixed and immobilized on poly-L-lysine coated coverslips.

### Optical measurement of cellular volume and area

DIC microscopy is carried out by illuminating the sample of interest with orthogonally polarized co-propagating wave fronts separated by a distance approximately equal to half the wavelength of the light source. These distinct wave fronts are generated by a Wollaston prism in combination with a polarizer placed in the illumination optics of the microscope. Image contrast is produced by specimen mass density variations that give rise to relative phase distortions in the transmitted orthogonally polarized wave fronts exiting the sample. A second Wollason prism and polarizer are used to carry out polarization-dependent common path interferometry; to interfere the exiting orthogonally polarized fronts from the diffraction limited imaging volume of the objective lens. This process converts sample induced phase perturbations in the orthogonal polarization modes into a detectable intensity (Preza et al., 2011). High numerical aperture (NA = 0.9) Köhler illumination enables the visualization of distinct transverse planes of the specimen along the optical axis.

**Figure 1:**
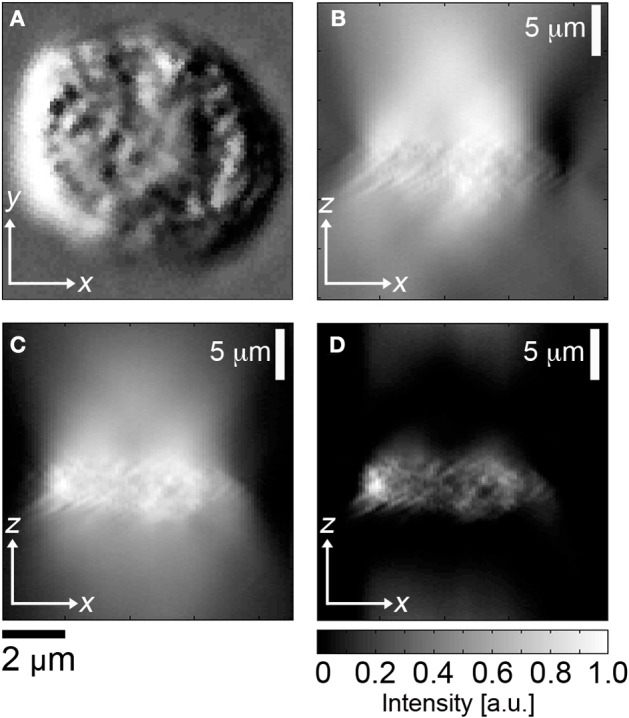
**Image segmentation of DIC image cubes. (A)** *En face* DIC image, leukocyte, **(B)** corresponding cross sectional image through center of cell, **(C)** Hilbert transform of **(B)**, **(D)** high pass filter applied to **(C)**.

HD-CTCs were relocated and through-focus DIC imagery at ×63 magnification, NA = 1.4, of each cell type was performed on a Zeiss Axio Imager 2 microscope (Carl Zeiss MicroImaging GmbH, Germany) under software control by SlideBook (Intelligent Imaging Innovations, Denver, CO), Figures 1A,B. Images were post-processed using a custom program written in MATLAB (The MathWorks, Inc., USA). Post-processing consisted of the application of a Hilbert transform to each *en face* DIC image to ensure optimal contrast in image cube construction (Arinson et al., 2000), Figure 1C. This process enables thresholding of DIC images at the cost of introducing image blur along the axial direction. Image blur is eliminated using a high-pass filter applied to each cross-sectional image of the image cube, Figure 1D.

The cross sectional areas of the cell in distinct sagittal planes separated by 0.5 [μm] were added together to obtain cellular volume. Each voxel in the analysis corresponds to a diffraction limited volume of 0.28 [μm] × 0.28 [μm] × 0.35 [μm] = 0.029 [fL]. No thresholding of the high-pass filtered Hilbert transformed sagittal images was required. Further details on the method and validation of the Hilbert DIC method for volume are reported in Baker et al. (2012). Cellular area was determined by outlining each cell in *en face* DIC images.

### The DIC image contrast arises from sub-cellular density gradients along the shear direction of the wollaston prisms

Following (Preza et al., 1999) we demonstrate that the DIC contrast is dominated by mass-density gradients inside the focal volume of the objective lens. This provides the rationale to adapt the language of “mass density variations” as opposed to “pathlength changes” in describing the origin of DIC image contrast.

We begin with Equation 20 of Preza et al. (1999); a three dimensional DIC imaging model that presumes temporal coherence of the waves interacting with the sample. The temporal coherence assumption is appropriate as the coherence length of the mercury lamp is on the order of cm while the thickness of the sample times the refractive index is on the order of μm. To determine the form of the fields interfering at the detector plane the amplitudes of the waves must be added together.

Referring to Figure 7 of Preza et al. (1999) we define our notation: Let A denote the area of the front focal plane of the condenser lens, $\vec{\xi }$ denote a point in the front focal plane of the condenser lens, $s(\vec{\xi })$ denotes the intensity of the light source in the focal plane of the of the condenser lens. *x*, *y*, *z* are coordinates in the image plane; *x*′, *y*′, *z*′ are coordinates in the object plane. The transmitted field of the specimen is denoted as *f*(*x*, *y*, *z*). The point spread function (PSF) of the DIC microscope is denoted *h*(*x* − *x*′, *y* − *y*′, *z* − *z*′). Lastly, the Köhler illumination plane wave fields are denoted $U_{k}(\vec{\xi },x,y)$; see the definition of these waves just after Equation 3 of Preza et al. (1999).

The three dimensional DIC imaging model is Equation 20 of Preza et al. (1999).

(1) $$i\left(x,\;y,\;z\right)=\int_{A}s(\vec{\xi })\text{​}|\int_{{\mathbb{R}}^{3}}f\left(x\prime ,\;y\prime ,\;z\prime \right)h\left(x-x\prime ,\;y-y\prime ,\;z-z\prime \right){U_{k}(\vec{\xi },\;x\prime ,\;y\prime )dz\prime dx\prime dy\prime |}^{2}d\vec{\xi }$$

We make the following simplifying assumptions:

1. We assume the DIC PSF is a delta function in *x* and *y* coordinates but we retain broadening along the optical axis. The DIC PSF is given in Equation 1 of Preza et al. (1999). The axial PSF is denoted *P*(*z* − *z*′). Δθ is the bias retardation introduced by the translation of the Wollaston prism. Δ*x* is the lateral shear introduced by the Wollaston prism. The DIC PSF is then
  (2) $$h\left(x-x\prime ,\;y-y\prime ,\;z-z\prime \right)=(\frac{1}{2}e^{-i\Delta \theta }\delta \left(x-x\prime -\Delta x,\;y\right)-\frac{1}{2}e^{i\Delta \theta }\delta \left(x-x\prime +\Delta x,\;y\right))P\left(z-z\prime \right)$$
2. The specimen is a “phase” object. The transmitted field is of the form
  (3) f(x′, y′, z′)=exp (−iϕ (x′, y′, z′))

Plugging in Equation 2 and 3 into Equation 1, we carry out the integration in the *x*′, *y*′ variables to obtain:

(4) $$i\left(x,\;y,\;z\right)\;=\frac{1}{4}\int_{A}s(\vec{\xi }){\left|\int_{\mathbb{R}}P\left(z-z\prime \right)\{U_{k}^{-}e^{-i\phi^{-}-i\Delta \theta }-U_{k}^{+}e^{-i\phi^{+}+i\Delta \theta }\}dz\prime \right|}^{2}d\vec{\xi }$$

In this expression we have utilized a shorthand notation in which $U_{k}^{\pm }=\;U_{k}(\vec{\xi },x\pm \Delta x,y,z\prime )$, and ϕ^±^ = ϕ(*x* ± Δ*x*, *y*, *z*′).

We next linearize the phase terms in Equation 4 by taking the small angle approximation of the complex exponentials; appropriate for weak index contrast specimens.

(5) $$i\left(x,\;y,\;z\right)=\frac{1}{4}\int_{A}s(\vec{\xi })|\int_{\mathbb{R}}P\left(z-z\prime \right)\{\left(U_{k}^{-}-U_{k}^{+}\right){+i(\phi^{+}U_{k}^{+}-\phi^{-}U_{k}^{-})-i\Delta \theta (U_{k}^{-}+U_{k}^{+})\}dz\prime |}^{2}d\vec{\xi }$$

Next, we assume that for fixed $\vec{\xi }$ that *U*_*k*_ ≈ *U*^−^_*k*_, *U*_*k*_ ≈ *U*^+^_*k*_, as these fields are separated along the shear direction by approximately λ/2. This enables the simplification of Equation 5 to:

(6) $$i\left(x,\;y,\;z\right)=\frac{1}{4}\int_{A}s(\vec{\xi })|{\int_{\mathbb{R}}P\left(z-z\prime \right)\left\{(\phi^{+}-\phi^{-}-2\Delta \theta )U_{k}\right\}dz\prime |}^{2}d\vec{\xi }$$

Multiplying and dividing the phase difference by the magnitude of the shear, Δ *x* = *s* we obtain:

(7) $$i\left(x,\;y,\;z\right)=\frac{1}{4}\int_{A}s(\vec{\xi })|U_{k}{\int_{\mathbb{R}}P\left(z-z\prime \right)\left\{\left(s\hat{x}\cdot \nabla \phi -2\Delta \theta \right)\right\}dz\prime |}^{2}d\vec{\xi }$$

We now develop an expression for the phase in terms of the mass-density of the sample.

Let $\vec{p}(z\prime ,\vec{\xi })$ denote the parameterized path of the wave. We utilize the variable dependence of the Kohler waves on $\vec{\xi }$ as described in Figure 7 of Preza et al. (1999). We presume that waves traverse the sample in straight-line trajectories—an assumption that is appropriate to weak index contrast samples such as cells. Let *f*_*c*_ denote the focal length of the condenser lens.

(8) $$\vec{p}(z\prime ,\;\vec{\xi })=z\prime \left(\frac{\xi_{x}}{f_{c}}\sqrt{1-\frac{\xi_{y}^{2}}{f_{c}^{2}}}\hat{x}+\frac{\xi_{x}\xi_{y}}{f_{c}^{2}}\hat{y}+\sqrt{1-\frac{\xi_{x}^{2}}{f_{c}^{2}}}\hat{z}\right)=z\prime \hat{\xi }$$

Letting *k* denote the wave number, ϕ the phase of waves traversing a specimen, and *n* the refractive index of the sample, under the paraxial approximation for weak index contrast specimens the phase of plane waves traversing the sample is given by:

(9) $$\phi \left(\vec{p}(z\prime ,\vec{\;\xi })\right)=k\int_{0}^{z\prime }\text{​}n\left(\vec{p}(z\prime \prime ,\vec{\;\xi })\right)dz\prime \prime .$$

The refractive index, as shown in Barer (1952) is related to the mass-density, denoted C [pg/fL], according to:

(10) $$n\left(x,\;y,\;z\right)=n_{o}+\;\alpha C\left(x,\;y,\;z\right).$$

Where *n*_*o*_ is the background index of the cell within a diffraction limited volume, α is the refractive increment ~ 0.2 [fl/pg]. We substitute Equation 10 into Equation 9 and then substitute the resulting expression into Equation 7 to obtain:

(11) $$i(x,\;y,\;z)\;=\frac{1}{4}\int_{A}\text{​}s(\vec{\xi })|U_{k}\alpha ks\hat{x}\cdot \nabla \int_{\mathbb{R}}P\left(z-z\prime \right)\int_{0}^{z\prime }\text{​}C\left(z\prime \prime \hat{\xi }\right)dz\prime \prime {dz\prime -2\Delta \theta U_{k}\int_{\mathbb{R}}P\left(z-z\prime \right)dz^{\prime }|}^{2}d\vec{\xi }.$$

Lastly, we note that the PSF limits the axial contributions of the mass density to axial locations near the focal position given by *z*. Without loss of generality, we presume an axial PSF with a square function shape with width 2Δ*z* about the focal point *z*. We find that:

(12) $$\int_{\mathbb{R}}P\left(z-z\prime \right)\int_{0}^{z\prime }C\left(z\prime \prime \hat{\xi }\right)dz\prime \prime dz\prime \approx \int_{z-\Delta z}^{z+\Delta z}C\left(z\prime \prime \hat{\xi }\right)dz\prime \prime .$$

Substituting Equation 12 into Equation 11 we arrive at the main result

(13) $$i(x,\;y,\;z)=\frac{1}{4}\int_{A}s(\vec{\xi })|U_{k}\alpha ks\int_{z-\Delta z}^{z+\Delta z}\hat{x}\cdot \nabla C\left(z\prime \prime \hat{\xi }\right)dz\prime \prime {-4\Delta \theta \Delta zU_{k}|}^{2}d\vec{\xi }.$$

This formula demonstrates that the DIC intensity contrast at a particular *z* location *inside* the sample is dominated by the spatial gradient of the concentration of cellular mass in that plane (the integral over *dz*″).

The “pathlength” point of view of DIC microscopy—while entirely valid—does not speak to the main contribution of contrast inside the cell. The axial distance over which the phase is being compared (the limits of integration of the axial variable *z*″) among the sheared trajectories is held fixed by the objective lens. Hence the height of the sample has been decoupled from the refractive index due to optical sectioning by the objective lens.

This point of view breaks down when the axial extent of the sample changes inside the focal volume: a situation that might arise as the focal volume first encounters the top of the cell.

### Optical determination of sub-cellular density fluctuation and spatial correlation

The DIC intensity is a non-linear function of the mass density gradient of the sample along the shear direction of the Wollaston prisms (Preza et al., 2011). Furthermore, the DIC intensity depends on the bias settings of the Wollaston prisms and illumination conditions (Preza et al., 2011). These complications often limit the use of DIC microscopy to qualitative investigations of morphology as the DIC intensity is difficult to relate to physical properties (e.g., density) of the sample. DIC microscopy images do, however, have a simple physical interpretation: they visualize edges of sub-cellular features. The variations of the DIC intensity in space can then be utilized, by analogy with time series analysis of random processes (Wainstein and Zubakov, 1962; Ishimaru, 1978; Subramanian et al., 2009), to quantify the average size of sub-cellular constituents, through spatial correlation, and the average magnitude of density fluctuations, through the analysis of DIC intensity amplitude variations. This heuristic analysis is able to probe the organization of cellular features ranging from the diffraction limit up to multiple microns in scale.

**Figure 2:**
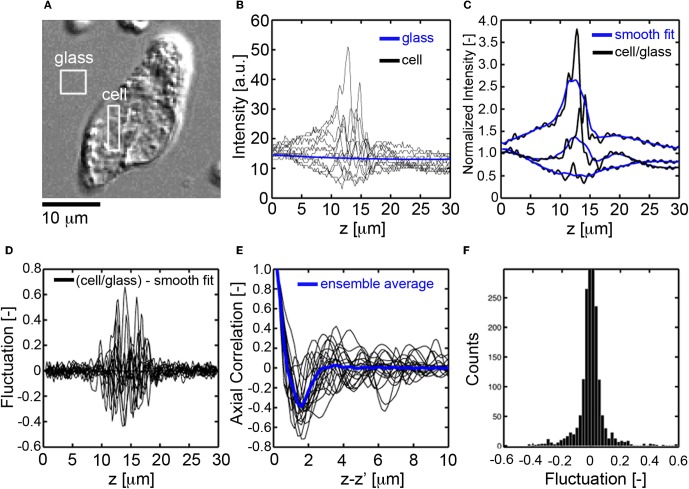
**Fluctuation and spatial correlation analysis of axial DIC intensity profiles. (A)** *En face* DIC image CTC. **(B)** Axial profiles of DIC intensity for pixels in the cell (black) and glass substrate (blue), **(C)** normalized DIC intensity profile (black, cell signal divided by glass signal) for pixels inside the cell and corresponding smooth fit (blue), **(D)** fluctuating part of the DIC intensity (normalized signal minus smooth fit), **(E)** autocorrelation function of the fluctuating part of the DIC signal, the correlation length *L*_*c*_[μm] is defined as the first zero crossing, **(F)** histogram of the fluctuating part of the DIC intensity, the mass-density fluctuations are quantified through the standard deviation, σ_*A*_[−], of the histogram.

For each pixel location (*x*, *y*) in the image of a cell (Figure 2A), the axial profile of the DIC intensity was recorded using a charge coupled device camera (CCD), (Figure 2B). The DIC signal was then normalized by the background glass signal to eliminate the effectsctc of exposure time and gain settings (Figure 2C). A smooth fit to the normalized DIC axial profile was then performed, (Figure 2C), and subtracted from the normalized DIC intensity to determine the fluctuating part of the DIC axial profile, (Figure 2D). The autocorrelation of the fluctuating part of the DIC signal was then numerically computed, (Figure 2E). The correlation length, *L*_*C*_ [μ m], for each axial profile within a cell was defined as the first zero-crossing of the autocorrelation function. The average magnitude of sub-cellular density fluctuations was assessed by binning the fluctuating part of the DIC intensity into a histogram, (Figure 2F). The standard deviation of the fluctuations, σ_*A*_ [−], was determined and recorded for each pixel location within the cell. Example *L*_*C*_ and σ_*A*_ maps are presented for CTCs and leukocytes in Figures 4C,D, respectively.

### Statistical analysis

The Jarque–Bera test was used to evaluate normality of all parameters. One-Way analysis of variance with Bonferonni *post-hoc* analysis was used to assess statistical significance among parameters across multiple normally distributed cell parameters. The Kruskal–Wallis test was used to assess significance among non-normally distributed parameters. *P*-values of 0.05 or less were considered significant. All quantities presented as mean ± standard deviation unless otherwise noted.

## Results

### Breast cancer associated HD-CTCs have larger volumes and areas than normal blood cell subpopulations

To investigate the validity of the optical volume measurement technique, we performed measurements on polystyrene spheres and found the measured volumes to coincide with the manufacturers specifications (Baker et al., 2012). To establish the ability of the technique to work with biological specimens, we purified populations of PLTs (*N* = 30) and RBCs (*N* = 20) from healthy volunteers. PLTs and RBCs were measured to have volumes (mean ± standard error of the mean) of 10.5 ± 0.5 fL, 100.6 ± 4.0 fL, within physiological norms determined by the Coulter method (Paulus, 1975; Lichtman, 2005). See Table 1.

**Table 1:** **Biophysical properties of normal peripheral blood cells and breast cancer associated HD-CTCs**.

**Cell type**	**Area [μm^2^]**	**Volume [fL]**	***L*_*C*_ [μm]**	**σ_*A*_[−]**	***N***
PLT	7.5 ± 0.3	10.5 ± 0.5	0.42 ± 0.03	0.06 ± 0.01	30
RBC	42.0 ± 1.0	100.6 ± 4.0	1.19 ± 0.05	0.06 ± 0.01	20
Leukocyte	48.0 ± 0.6	234.1 ± 4.1	0.87 ± 0.03	0.17 ± 0.05	100
HD-CTC	135.6 ± 6.0^*^	851.6 ± 45.8^*^	0.80 ± 0.04^*^	0.12 ± 0.04^*^	42

* Denotes p < 0.001 with respect to leukocytes. Leukocytes and HD-CTCs are from the same patient while PLTs and RBCs were collected from healthy donors.

**Figure 3:**
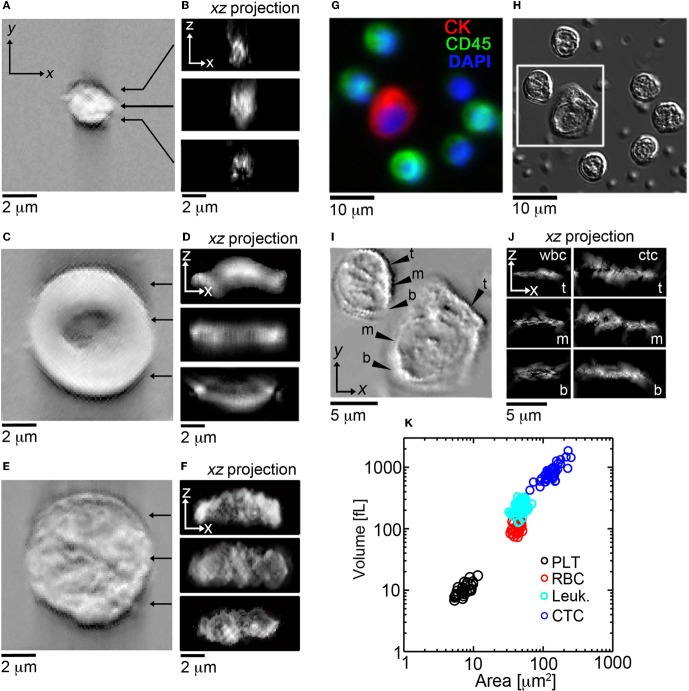
***En face* and cross sectional post-processed DIC images of normal blood cell populations and HD-CTCs.** Representative *en face* and sagittal Hilbert transformed DIC images of **(A,B)** PLT, **(C,D)** RBC, **(E,F)** leukocytes. **(G)** Merged fluorescence image of HD-CTCs and leukocytes from breast cancer patient, **(H)** corresponding DIC image, **(I)** corresponding Hilbert transformed DIC image, **(J)** corresponding cross sectional images at the t, top; m, middle; b, bottom locations denoted in **(I)**. **(K)** Scatter plot of cell areas vs. corresponding volume for each cell type.

After this initial validation of the technique, we set out to determine if HD-CTCs had distinct volumes from leukocytes. We measured the volumes of HD-CTCs (*N* = 42, using four slides from the different blood draws of one patient) and leukocytes (*N* = 100) identified in the peripheral blood of a breast cancer patient with known metastatic disease, (Figure 3). Leukocytes were chosen at random in the field of view containing the HD-CTC. HD-CTCs were determined to have significantly larger volumes (mean ± standard error of the mean), 851.6 ± 45.8 [fL], than leukocytes, 234.1 ± 4.1 fL, *p* < 0.0001 (Table 1).

Next, we measured the area of each cell type to test for overlap of this parameter among HD-CTCs and leukocytes as previously observed in colorectal cancer (Marrinucci et al., 2009a) and prostate cancer (Stott et al., 2010). The area for normal cell types was measured to be 7.5 ± 0.3 [μm^2^] (PLT), 42.0 ± 1.0 [μ m^2^] (RBC), 48.0 ± 0.6 [μm^2^] (leukocytes). CTCs were found to have a mean area of 135.1 ± 6.0 [μm^2^], significantly larger than leukocytes, *p* < 0.0001 (Table 1).

### HD-CTCS exhibit smaller mass-density fluctuations and shorter-range mass-density spatial correlations in comparison to leukocytes

Previously, we determined by Wright-Giemsa staining that CTCs have a high degree of pleomorphism, exhibit a range of high and low nuclear-to-cytoplasmic ratios, and possess morphological features similar to the primary and/or metastatic lesions in breast (Marrinucci et al., 2007), colorectal (Marrinucci et al., 2009a), and lung (Marrinucci et al., 2009b) cancer.

As the organization of cellular mass density is central in determining the absorption properties exhibited by stained cells and thus central to the qualitative evaluation of CTCs by pathologists, we sought to quantify the heterogeneity of sub-cellular mass density using the optical sectioning and edge detection capabilities of DIC microscopy. DIC intensity variations along the optical axis were used to infer the relative magnitude of sub-cellular density fluctuations, σ_*A*_ [−], and spatial correlations along the axial direction can be used to assess the average size of sub-cellular constituents, *L*_*C*_ [μm].

First, we established the utility of the (σ_*A*_, *L*_*C*_) parameters in distinguishing purified populations of normal blood cells, (Figures 5B,C). *L*_*C*_ was found to be unique to each blood cell type: 0.42 ± 0.03 [μm] (PLT), 1.19 ± 0.05 [μm] (RBC), 0.87 ± 0.03 [μm] (leukocyte), *p* < 0.001, while σ_*A*_ values were identical for PLTs and RBCs: 0.06 ± 0.01 [−], though distinct for leukocytes: 0.17 ± 0.05 [−], *p* < 0.001, (Table 1).

**Figure 4:**
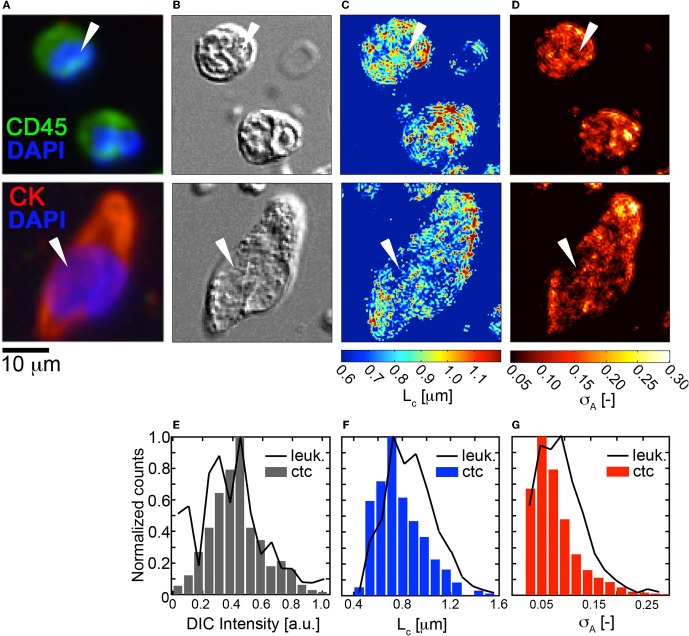
**Fluctuation and spatial correlation maps: comparison of HD-CTCs and leukocytes. (A)** Merged fluorescence image of HD-CTCs and leukocytes, **(B)** corresponding DIC images, **(C)** spatial correlation length map, **(D)** density amplitude fluctuation map, **(E)** histogram of DIC intensity of cells indicated with white arrows in **(B)**. **(F)** HD-CTC and leukocyte *L*_*C*_ histograms of cells indicated in **(C). (G)** HD-CTC and leukocyte σ_*A*_ histograms of cells indicated in **(D)**.

Next, we explored the (σ_*A*_, *L*_*C*_) properties of HD-CTCs and compared these to leukocytes. Mapping of the σ_*A*_ and *L*_*C*_ parameters over the nuclear and cytoplasmic compartments of the cell revealed that CTCs had reduced nuclear density fluctuations and reduced nuclear spatial correlation lengths in comparison to leukocytes, (Figures 4C,D). Upon binning the (σ_*A*_, *L*_*C*_) values corresponding to each cell type into histograms, systematic decreases in both of these parameters were observed cell-wide for CTCs in comparison to leukocytes, (Figures 4F,G). No differences between HD-CTCs and leukocytes were observed in histograms of the DIC image alone, (Figure 4E).

**Figure 5:**
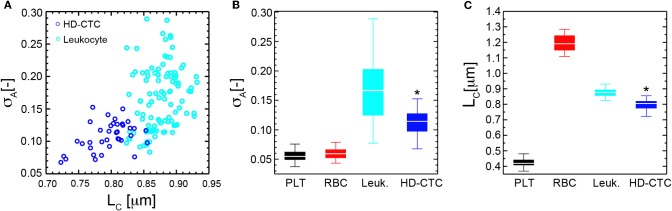
**Mass density fluctuation and spatial correlation metrics for normal blood cell subpopulations and HD-CTCs. (A)** Scatter plot of spatial correlation length vs. amplitude fluctuations for HD-CTCs and leukocytes from breast cancer patient. **(B)** Box plot comparing population averages of the axial resolved DIC amplitude fluctuations averages of the axial resolved DIC intensity spatial correlation length among PLTs, RBCs, leukocytes, and HD-CTCs. **(C)** Box plot comparing population averages of the axial resolved DIC intensity spatial correlation length among PLTs, RBCs, leukocytes, and HD-CTCs. ^*^Denotes *p* < 0.001 in comparison to leukocytes.

To determine the ability of the (σ_*A*_, *L*_*C*_) parameters to quantitatively characterize CTCs and leukocytes, we computed the mean values of σ_*A*_ and *L*_*C*_ for CTCs (*N* = 42) and compared these to leukocytes and found statistically significant decreases in both parameters among HD-CTCs in comparison to leukocytes, (Figure 5, Table 1). The mean subcellular constituent size, *L*_*C*_, for HD-CTCs was 0.80 ± 0.04 [μm] compared to 0.87 ± 0.03 [μm] for leukocytes, *p* < 0.001; density fluctuations for HD-CTCs, σ_*A*_, were found to be 0.12 ± 0.04 compared to 0.17 ± 0.05 for leukocytes, *p* < 0.001, Table 1.

## Discussion

Clinical studies have demonstrated that metastatic cancer is accompanied by the presence of CTCs in the peripheral circulation across the major carcinomas (Allard et al., 2004) and that CTCs possess morphologic similarities to primary and/or metastatic lesions (Marrinucci et al., 2007, 2009a,b) and are morphologically distinct from the surrounding white blood cell population (Marrinucci et al., 2012).

To date, the biophysical characterization of CTCs has been restricted to two-dimensional investigations of morphology, area, and nuclear to cytoplasmic ratio. Here we quantified basic three-dimensional biophysical properties of CTCs associated with metastatic disease of the breast: volume and mass density variations. We find that HD-CTCs are characterized by larger volumes, decreased mass-density fluctuations, and possess shorter-range spatial density correlations in comparison to leukocytes. We attribute this basic difference in HD-CTCs and leukocytes to the high nuclear content of leukocytes, giving rise to increased amplitude fluctuations, and the increased amount of compaction of nuclear material in leukocytes, giving rise to larger spatial correlations (sub-cellular constituent sizes). These results mirror the qualitative observation that HD-CTCs in this study had large, spread out nuclei, that were homogenous in comparison to the surrounding leukocytes, Figure 4B. Future studies linking DIC based measurements of mass density variations with nano-scopic tools (Subramanian et al., 2008) will provide insight into nuclear architecture over the nano-to-micro scale divide (Zink et al., 2004).

The optical sectioning capability of DIC microscopy provides a means to account for structural information that is normally out of focus or not detectable in fluorescence and stain based imaging, thus yielding a complementary characterization of CTC cellular structure. Indeed, we found that in some instances the cell bodies of CTCs were spread across multiple planes perpendicular to the optical axis, spanning up to 10 μm, while the cell bodies of the leukocyte population were consistently confined to a 3–4 μm range about the central focal plane. As previous reports (Marrinucci et al., 2009a; Stott et al., 2010) utilizing fluorescence microscopy have documented the general separation but partial overlap of CTC area with leukocyte area and the similarity of nuclear to cytoplasmic ratio among breast cancer associated CTCs and leukoctyes (Marrinucci et al., 2007) the future measurement of CTC volume across tumor type and disease stage will provide further insight into the geometric similarities and differences of CTCs and the corresponding leukocyte population.

Physical parameters independent of volume and area, such as density amplitude fluctuations and spatial correlations introduced in this study, provide a complementary measurement of cellular structure that attempt to quantify the qualitative observations of pathologists using stain based analysis under light microscopy. Investigations on transformed human cell lines utilizing label-free optical scattering and spectroscopic reflection microscopy measurements have demonstrated the presence of distinct optical signatures from cancer cells: increased sub-cellular constituent size (Mourant et al., 1998), and an increased amount of structural “disorder” at the nanoscale (Subramanian et al., 2008). These observations suggest that cancer at the cellular level is characterized by unique structural features that can be utilized to detect, monitor, and potentially understand cancer.

The DIC method presented is utilized subsequent to molecular based identification of CTCs using the HD-CTC assay. Future validation studies are required to assess the sensitivity and specificity of the biophysical metrics in relation to the HD-CTC inclusion criteria. To be competitive with the HD-CTC assay, the DIC method would need to be drastically sped up. Currently, the DIC method requires two minutes to complete image cube acquisition of a single field using a Zeiss Axio Imager 2 with a moveable stage. This time could be reduced by a factor of 10 with a piezo-driven objective lens holder. Post-processing of image cubes in MATLAB currently takes 5–7 min on a Dell TerraStation using the full resolution image cubes. Down sampling of the cubes to courser grids could speed up computation time at the possible expense of losing information. Future optimization studies are required to make the label-free DIC method competitive with label-based approaches.

Probing the “fluid phase” of cancer has been technologically challenging owing to the minute concentration of CTCs in the peripheral blood of cancer patients with metastatic disease. The advent of modern CTC isolation and characterization methods has recently enabled the use of these rare cells to survey primary and metastatic tumors through non-invasive blood draws. This study suggests that CTCs may possess a distinct set of physical parameters in comparison to the white blood cell populations in the same patient. However, these parameters have yet to be developed completely to demonstrate their utility for clinical applications. Further studies will be required across multiple patients and disease types to determine whether the physical parameters observed in this study are conserved across patient populations, time, treatment, and cancer type. Measurement of the basic biophysical characteristics of CTCs is critical to both understanding the physical characteristics and chemical kinetics of metastasis and aiding in future detection of CTCs in non-perturbative ways to maintain the viability of these enigmatic cells.

### Conflict of interest statement

The HD-CTC technology has been licensed to Epic Sciences. Authors of this manuscript have ownership in Epic Sciences.
